# Effect of Auricular Acupressure on Uremic Pruritus in Patients Receiving Hemodialysis Treatment: A Randomized Controlled Trial

**DOI:** 10.1155/2015/593196

**Published:** 2015-10-01

**Authors:** Cui-na Yan, Wei-guo Yao, Yi-jie Bao, Xiao-jing Shi, Hui Yu, Pei-hao Yin, Gui-zhen Liu

**Affiliations:** Putuo Hospital, Shanghai University of Traditional Chinese Medicine, Shanghai 200062, China

## Abstract

*Background*. Uremic pruritus (UP) is a common symptom in patients undergoing maintenance hemodialysis for end-stage renal disease (ESRD).* Objective*. To determine the clinical efficacy of auricular acupressure therapy on pruritus in hemodialysis patients and to explore possible underlying mechanisms.* Methods*. Patients receiving maintenance hemodialysis at a referral medical center were recruited and assigned to intervention (*n* = 32) and control (*n* = 30) groups. The intervention group underwent auricular acupressure treatment three times a week for six weeks. Auricular acupressure was not applied to patients in the control group. However, tape without* Vaccaria* seeds was applied to the same six auricular acupoints as the intervention group. Pruritus scores were assessed using VAS scores, and enzyme-linked immunosorbent assays (ELISA) were used to measure levels of other possible contributory biochemical factors.* Results*. There was a significant difference in mean VAS scores between the postintervention and control groups during follow-up (3.844 ± 1.687 versus 5.567 ± 2.285, *F* = 22.32, *P* < 0.0001). Compared to the control group, serum histamine levels in the postintervention group at the six-week follow-up had decreased significantly (*F* = 5.01, *P* = 0.0290).* Conclusion*. Our findings suggest that auricular acupressure may be a useful treatment in the multidisciplinary management of UP in ESRD patients.

## 1. Introduction

Uremic pruritus (UP) is a common symptom in patients with end-stage renal disease undergoing maintenance hemodialysis (HD) [1]. UP or itching affects about 20 to 50 percent of patients with ESRD [2–4] with no other primary skin diseases or systemic or psychological dysfunctions that might cause pruritus. Moreover, while the distribution of pruritus between patients is highly variable, its symmetrical manifestation is a common feature [1].

The pathogenic molecular basis of pruritus in chronic renal failure remains elusive, which limits options for effective treatment. Previous studies showed that, besides metabolic factors and dialysis clearance, inflammation is the factor mainly associated with UP in patients receiving hemodialysis [5]. Aoki et al. reported that the skin of patients with ESRD and UP had increased numbers of mast cells; these cells release a variety of substances including histamine, tumor necrosis factor (TNF-a), and interleukin-6 (IL-6), which are common inflammatory markers [6]. Unfortunately, the results of different studies are inconsistent, and most of the described treatments have had limited success [7].

Pruritus is frequently refractory to treatment and is associated with substantial medical, psychological, and social disturbances in patients receiving hemodialysis [8]. Current UP treatments include oral antihistamines, gabapentin, ondansetron, thalidomide, naltrexone/nalbuphine, ultraviolet (UV) light, and topical tacrolimus [9]. However, many nonpharmacological treatment methods such as acupressure are also used to relieve UP discomfort [10].

In traditional Chinese medicine, ear acupoints correspond to body organs and meridians, as well as the four limbs and skeleton; they serve both as a disease reaction point and treatment trigger. Auricular acupressure is the combination of visceral state doctrine and meridian theory; it can be used to adjust different parts of the human body by reconditioning the meridians, conductivity of senses, and energy deficiency and excess to achieve a relative balance and facilitate therapeutic modalities that target disease [11]. Auricular acupressure therapy combines a treatment modality with traditional Chinese medicine. It is safe and effective, with fewer side effects, and is more easily accepted by patients. The present study evaluated the efficacy of auricular acupressure in patients receiving hemodialysis treatment for UP. We hypothesized that at the end of the six-week intervention period patients in the auricular acupressure group would have a greater reduction in UP scores and more improvements in health-related quality of life than those measures in the control group.

## 2. Materials and Methods

### 2.1. Patients

**Figure 1:**
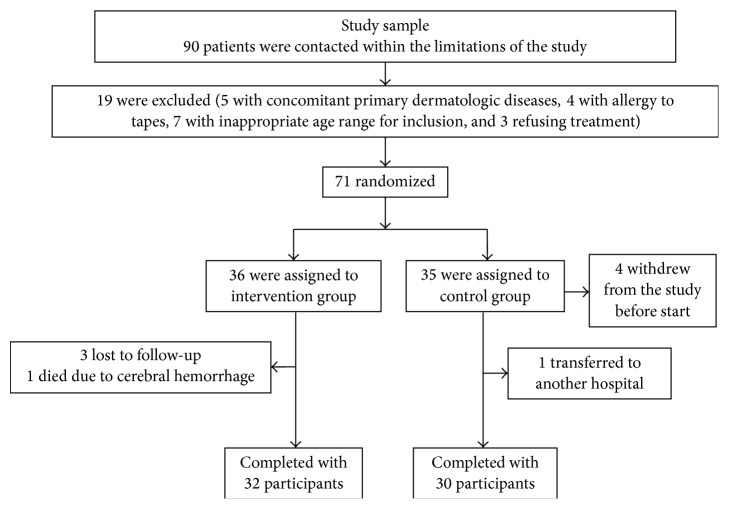
Participant screening, randomization, and completion in the six-week intervention study.

Patients were referred by doctors and acupuncturists in the Department of Hemodialysis in Putuo Hospital, part of the Shanghai University of Traditional Chinese Medicine. The current study took place between April and October 2014 and included 32 and 30 participants in the intervention and control groups, respectively (Figure 1). The study procedures, risks, benefits, and data management were clarified in detail before the patients were asked to provide their informed consent.

All participants enrolled in this study were undergoing hemodialysis three times per week for 3-4 hours each visit, with a blood flow rate maintained above 200–250 mL/min to guarantee sufficient dialysis. Participants met the following inclusion criteria [10]: (1) between 20 and 65 years of age; (2) undergoing dialysis at HD units for 6 months; (3) having had pruritus for at least 3 months; (4) having at least 3 or more points on the visual analogue scale (VAS) for pruritus; (5) having sufficient cognitive ability to answer questions regarding outcome measures; (6) not having previously been diagnosed with skin disease involving pruritus; (7) not having previously been diagnosed with liver disease, psychiatric disorder, or cancer; (8) not having had any nervous, vascular, or soft-tissue disorders in their extremities; (9) having no visible infection or having undergone surgical operations on their extremities; and (10) having provided informed consent. Participants were permitted to continue their routine medications and maintain their usual visits to their primary care physicians or nephrologists throughout the study. No other nonpharmacological treatments were permitted at the pruritus sites for participants in both groups during the study period.

### 2.2. Procedure

**Figure 2:**
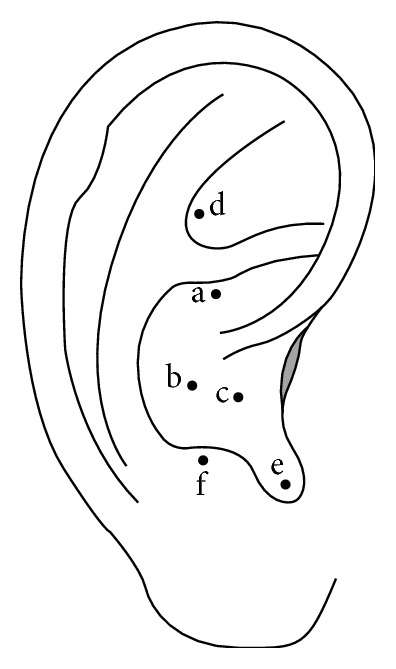
Location of auricular acupoints used in the auricular acupressure treatment group; a: the “kidney”: the back of the bottom ear helix (i.e., the concha 10 region, CO10); b: the “lung”: adjacent to the area of the “heart” and the “trachea” (i.e., the concha 14 region, CO14); c: the “heart”: in the middle ear cavity depression (i.e., the concha 15 region, CO15); d: the “Shenmen”: in the upper part of the posterior 1/3 of the triangular fossa (i.e., the triangular fossa 4 region, TF4); e: the “endocrine”: within the intertragic notch in the lower front concha (i.e., the concha 18 region, CO18); f: the “subcortical”: within the inner side of the tragus (i.e., the tragus 4 region, AT4).

The intervention group underwent* Vaccaria* seed alignment (Runshi Trading, China). Six auricular acupoints were selected for their potential effects on pruritus, including the “kidney” (CO10), the “lung” (CO14), the “heart” (CO15), “Shenmen” (TF4), the “endocrine” (CO18), and the “subcortical” (AT4); these organs are identified in traditional Chinese medicine and are distinct from organ sites in Western medicine (Figure 2).

The auricular acupressure protocol was as follows: the ear point was disinfected with 75% alcohol, the most sensitive point was identified, and pressure was applied at the point to mark the skin surface. A* Vaccaria* seed was attached and pressure was applied to each ear point for 1-2 min with appropriate finger force until the patients felt a tolerable soreness, numbness, and heat. All auricular acupressure operators were medical staffs with professional backgrounds; they were also responsible for collecting subjective and objective data and outcomes before and after the intervention.

### 2.3. Intervention Group

The auricular acupressure intervention occurred three times weekly for six weeks. The patients were asked to press their acupoints 5–8 times a day, with one mandatory press before going to sleep every night. One side of the ear acupoint was chosen each time, alternating bilaterally. The tape was replaced every other day and removed every Sunday as a break day. The patients were informed that the tape should be protected against moisture and contamination in order to avoid loss of tape or auricular skin inflammation and that the tape should not be attached to damaged skin or skin with frostbite or other lesions in order to prevent infection. The baseline was defined as the VAS score collected during the face-to-face interview at week 0. The research team collected additional data from participants by repeating the VAS testing after participants had completed the full course of treatment.

### 2.4. Control Group

The control group did not undergo auricular acupressure treatment; there was no* Vaccaria* seed in the tape placed on the same six auricular acupoints as the intervention group, and the patients were told that the tape contained a traditional Chinese medicine that could reduce pruritus. The tape was replaced every other day and removed every Sunday as a break day. Participant information was collected identically to the intervention group, and VAS scores were determined through face-to-face interviews.

### 2.5. Outcome Measures and Follow-Up

#### 2.5.1. VAS

VAS scores [12] indicated the intensity and severity of pruritus experienced by the study participants: 0 meant no pruritus, while 10 indicated very intense pruritus. The scale, which has been used to assess pain intensity in many studies, has been shown to be a valid and reliable tool. It has also been used to assess the intensity and severity of pruritus, which is a subjective sensation like pain [13, 14].

#### 2.5.2. Blood Sample Collection

A 5-mL disposable vacuum syringe was used to sample 3 mL of blood. After 1 hour of standing and 15 min high-speed centrifuge at 3,000 rpm/min, the sample supernatant was stored at −80°C for enzyme-linked immunosorbent assay (ELISA) analysis. Samples were collected before hemodialysis was performed.

### 2.6. Biochemical Parameters

Serum concentrations of histamine, substance P, protease activated receptor-2 (PAR-2), and tryptase were measured by ELISA; all standards and samples were run in duplicate, following the manufacturer's instructions for commercial histamine (HA) (CEA927Ge, cloud-clone), substance P enzyme immunoassay (EIA) (583751, Cayman Chemical), PAR-2 (SEA852Hu, cloud-clone), and tryptase (TPS) (SEB070Hu, cloud-clone) kits. Serum calcium, phosphorus, and PTH concentrations were measured in the clinical laboratory of our hospital using standard techniques.

### 2.7. Ethics

This clinical study received formal approval from the ethics committee of Putuo Hospital, part of the Shanghai University of Traditional Chinese Medicine. The primary researcher explained the purpose of the study, data collection procedures, information confidentiality, and the right to withdraw from the study to prospective participants. Patients signed consent form if they agreed to participate in the study.

### 2.8. Randomizations and Procedures for Setting

In order to achieve comparable groups for known and unknown risk factors, a random allocation sequence was generated using randomized block methods in SAS 9.1.3 statistical software in a 1 : 1 allocation ratio and with block size = 4. The eligible participants were divided into control and intervention groups. The practitioners participating in the study did not take part in the randomization process, and the evaluation and analysis of results was performed by professionals blinded to the treatment assignments. These blinded professionals performed outcome assessments and checked for missing data. Except for the practitioners, all individuals involved in the outcome assessment were blinded to participant allocation.

### 2.9. Statistical Analysis

All tests were performed using GraphPad Prism Software 5.0 (GraphPad Software, La Jolla, CA) and SAS software, version 9.1.3 (SAS Institute Inc., Cary, NC, USA). The data were expressed as mean ± standard deviation (SD) and median (interquartile range).* t*-tests were used for comparison of independent groups involving normally distributed data, and Wilcoxon tests were used for comparison of independent nonnormally distributed groups. VAS scores and biochemical parameters in the intervention and control groups were compared separately according to their follow-up weeks with ANCOVA. *P* < 0.025 was considered statistically significant for the primary outcome (VAS score), while *P* < 0.05 was considered statistically significant for secondary outcomes.

## 3. Results

### 3.1. Patient Baseline Characteristics

Table 1 shows that the baseline data of the 62 participants were reasonably well balanced between the two groups. Age, gender, duration of dialysis, dialysis efficiency (Kt/V), urea reduction ratio (URR), body mass index (BMI), primary disease diagnosis, and VAS score were similar among participants in the intervention and control groups. The levels of calcium, phosphorus, PTH, histamine, substance P, PAR-2, and tryptase were also measured as biochemical factors that might contribute to pruritus. However, no significant differences in these baseline biochemical parameters were detected between the groups (*P* > 0.05).

### 3.2. Primary Outcome

**Figure 3:**
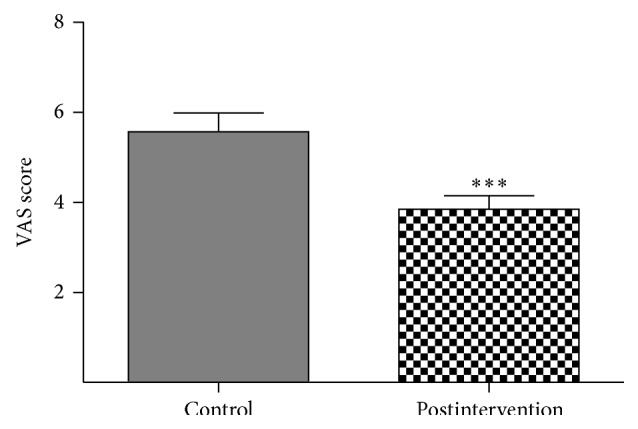
Mean change in visual analog scale (VAS) scores after six weeks, according to the postintervention group. Participant visual analog scale (VAS) scores indicate pruritus intensity and severity: 0 means no pruritus and 10 means very intense pruritus. ^*∗∗∗*^ *P* < 0.001 versus control group.

As shown in Table 1, the mean baseline VAS scores of the intervention and control groups were 5.750 ± 2.032 and 5.600 ± 2.127, respectively. These scores did not differ significantly between these groups (*P* > 0.05). There was a significant difference between the mean VAS scores of participants in the postintervention and control groups in their follow-up assessment (3.844 ± 1.687 versus 5.567 ± 2.285, *F* = 22.32, *P* < 0.0001) (Figure 3).

### 3.3. Mean Changes in Seven Secondary Outcomes after Six Weeks

**Figure 4:**
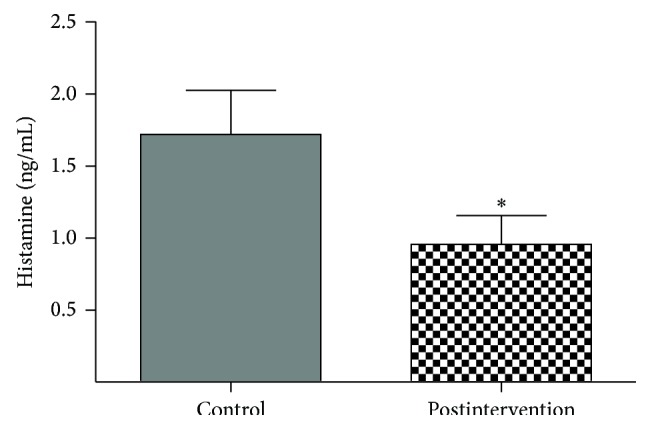
Mean change of serum histamine levels after six weeks, according to the postintervention group. ^*∗*^ *P* < 0.05 versus control group.

Baseline serum histamine levels (Table 1) did not differ significantly between the intervention and control groups (*P* > 0.05). However, comparison of the serum histamine levels at the six-week follow-up revealed a significantly greater decrease in the postintervention group (Figure 4) than the control group (*F* = 5.01, *P* = 0.0290).

**Figure 5:**
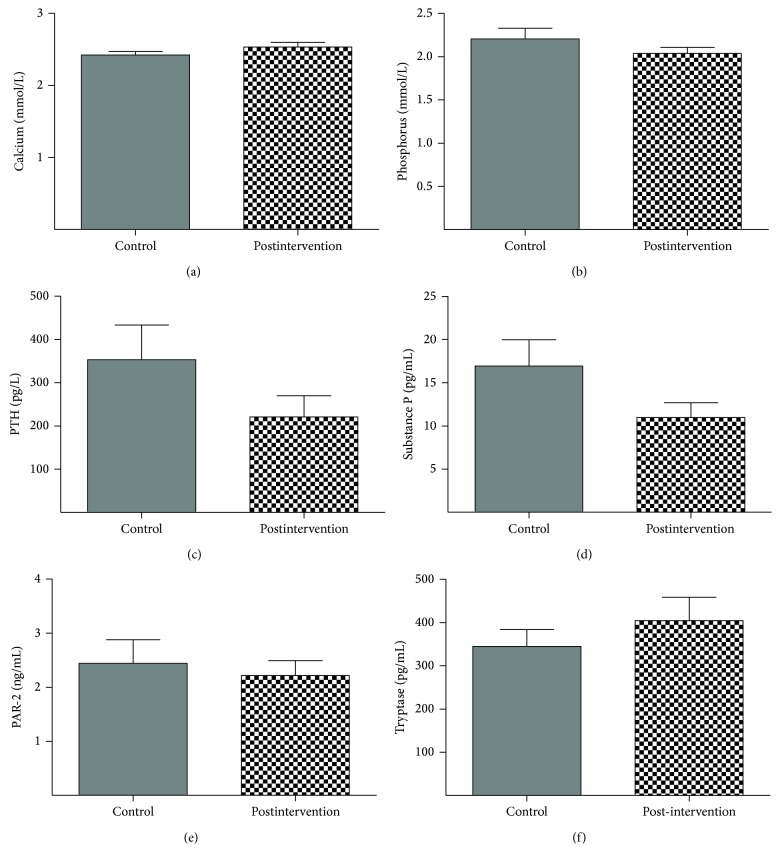
Mean changes in six secondary outcomes after six weeks, according to the postintervention group; *P* > 0.05 for postintervention group versus control group.

The levels of biochemical factors associated with pruritus, including calcium, phosphorus, PTH, substance P, PAR-2, and tryptase, did not differ significantly between groups (Figure 5). The six-week calcium serum levels for the postintervention and control groups were 2.532 ± 0.3651 mmol/L and 2.419 ± 0.2823 mmol/L, respectively (*F* = 1.68, *P* = 0.2001), and 2.038 ± 0.4012 mmol/L and 2.206 ± 0.6785 mmol/L for serum phosphorus levels, respectively (*F* = 1.57, *P* = 0.2145). The serum PTH levels were 119.5 (29.83, 268.3) pg/L and 234.0 (98.90, 435.5) pg/L, respectively (*F* = 2.02, *P* = 0.1609). Serum levels of substance P were 10.81 (4.336, 22.59) pg/mL and 9.332 (4.099, 14.50) pg/mL, respectively (*F* = 3.89, *P* = 0.0533). PAR-2 serum levels were 1.695 (1.010, 3.403) ng/mL and 1.713 (0.8283, 3.683) ng/mL, respectively (*F* = 0.16, *P* = 0.6925), and serum tryptase levels were 363.9 (205.0, 437.4) pg/mL and 305.2 (148.7, 447.0) pg/mL, respectively (*F* = 0.41, *P* = 0.5261).

No adverse events were noted during the study interventions.

## 4. Discussion

This experimental study was performed with 62 participants undergoing hemodialysis. As the primary outcome, the VAS scores of individuals in the postintervention group decreased over time compared to VAS scores in the control group (*P* < 0.001). Furthermore, serum levels of histamine in the postintervention group declined more significantly than those in the control group. These results of this study show that auricular acupressure is effective in reducing the frequency and severity of UP associated with hemodialysis, as well as improving quality of life.

Acupuncture is effective for other types of pruritus, including atopic dermatitis and asteatotic eczema; acupuncture has also been shown to have significant beneficial effects on histamine-induced itch in healthy volunteers. Pfab et al. [15] investigated the effect of acupuncture on type I hypersensitivity itch and skin reaction in a double-blind randomized placebo-controlled crossover trial, reporting that acupuncture resulted in a significant reduction in type I hypersensitivity itch in patients with atopic eczema.

Auricular acupressure therapy, a traditional Chinese medicine treatment modality, has the same purpose as acupuncture: simple, convenient, and cost-effective method widely used for clinical management of diseases [16–18]. The VAS scores of individuals in the postintervention group in our study decreased over time compared to scores in the control group (*P* < 0.001). Similarly, Akça et al. [10] showed that acupressure applied on acupuncture points using a transcutaneous electrical nerve stimulation (TENS) acupuncture apparatus was effective in reducing the frequency and severity of UP associated with hemodialysis. Meanwhile, Che-Yi et al. [19] reported lower mean posttrial pruritus in the group of participants who received acupuncture on the LI 11 acupuncture point, compared to both pretrial and control group scores. The treatment was reported to be successful after a 3-month follow-up period. Auricular acupressure for treatment of insomnia, hypertension, and depression in uremic patients has also been reported, which provides new insights for management of ESRD patients with UP.

UP develops for various reasons at any stage of treatment in patients receiving hemodialysis due to ESRD. Xerosis and hypohidrosis, the presence of pruritogenic cytokines (histamine, kallikrein, interleukin- (IL-) 2, acetylcholine, and other substances released by histamine-mediated mast cell stimulation), as well as secondary hyperparathyroidism, and immune-inflammatory reactions, have been proposed as reasons for the occurrence of UP [20, 21]. Multiple hypotheses and parameters have been put forth to explain the pathophysiology of UP; however, a consensus has not yet been reached in the literature [22].

The association of mast cells and UP in patients with ESRD has been extensively studied, which could possibly serve as one of the major pathogenic factors. Studies have shown that the skin of ESRD patients with UP has increased numbers of mast cells; these cells can release a variety of substances, including histamine, TNF, and IL, which are also common inflammatory markers [6]. The number of mast cells and histamine levels are reportedly higher in hemodialytic patients with pruritus compared to those in patients without pruritus symptoms [23]. Mast cells are an important class of immune cells that participate in a variety of biological responses through the release of particulate mediators in the body. They are involved in immune defense, endocrine regulation, and axonal reflex. In addition to hypersensitivity, mast cells also play a role in various immune functions, including innate and adaptive immunity. The major component of skin keratinocytes also plays an important role in pruritus pathogenesis, during which histamine released from mast cells serves as the major mediator [24].

For example, Kremer et al. [25] reported that intradermal application of histamine by iontophoresis or intradermal injection causes itching after a characteristic latency of up to 1 min, which is accompanied by a wheal and surrounding flare. Abundant mast cell clusters were found within acupoints in previous studies [26]. Our study also observed significantly reduced serum histamine levels in the postintervention group compared to the control group at the six-week follow-up (Figure 4) (*F* = 5.01, *P* = 0.0290). We believe that auricular acupressure decreases pruritus symptoms in patients undergoing sustained hemodialysis by reducing histamine levels.

However, histamine is present in both bound and inactive forms within tissue mast cell particles and basophils. When the body is subjected to physical and chemical irritation or allergic reactions, cell degranulation occurs, resulting in the release of histamine and subsequent biological effects. Acupuncture has a biphasic regulatory role for physiological and pathological conditions, and studies have shown that activation of acupuncture points can induce mast cell degranulation in localized connective tissue [27], and electric acupuncture can cause mast cells to gather at these points [28]. For example, auricular acupuncture on “stomach,” “Zusanli,” and other points can reduce visceral inflammation and ultimately suppress mast cell degranulation, which may play a therapeutic role in treatment of gastric ulcers [29]. Based on our data, the effect of auricular pressure in decreasing pruritus in hemodialysis patients is associated with reduced histamine levels (Figure 4). Auricular acupressure may control mast cell degranulation and consequently reduce the histamine release.

Analysis of mediators closely associated with mast cells revealed no significant differences in levels of substance P, PAR-2, and tryptase between the postintervention and control groups. This finding might be due to insufficient acupressure therapy duration or follow-up period. There were also no significant differences in calcium, phosphorus, and PTH levels between the groups; future multicenter randomized trials with larger sample sizes and longer treatment durations are necessary to verify the long-term efficacy of auricular acupressure among hemodialysis patients with pruritus.

The patients in the control group were informed that the tape contained a traditional Chinese medicine that could reduce pruritus. The tape was replaced every other day and removed every Sunday as a break day. This procedure was meant to eliminate the potential placebo response during the VAS interview and to prevent patients from identifying the control and intervention groups. The practitioners participating in this study did not take part in the randomization process, and evaluation and analysis of results was performed by professionals blinded to the assignation of treatment options; therefore, we consider the results of this study to be objective and reliable.

## 5. Conclusion

Our preliminary findings indicate that auricular acupressure may be a useful treatment in the multidisciplinary management of UP in patients with ESRD. Longer-term studies involving larger clinical samples are warranted to assess the generalizability of our findings and to deepen our understanding of this promising therapeutic approach.

## Supplementary Material

Enzyme-linked immunosorbent assay
1.Determine wells for diluted standard, blank and sample. Prepare 5 wells for standard points,1 well for blank. Add 50ul each of dilutions of standard(read Reagent Preparation), blank and samples into the appropriate wells, respectively. And then add 50ul of Detection Reagent A to each well immediately. Shake the plate gently. Cover with a Plate sealer. Incubate for 1 hour at 37℃. Detection Reagent A may appear cloudy.Warm to room temperature and mix gently until solution appears uniform.
2.Aspirate the solution and wash with 350ul of 1× Wash Solution to each well using a squirt bottle, multi-channel pipettle, manifold dispenser or autowasher, and let it sit for 1-2 minutes. Remove the remaining liquid from all wells completely by snapping the plate onto absorbent paper. Repeat 3 times. After the last wash, remove any remaining Wash Buffer by aspirating or decanting. Invert the plate and blot it against absorbent paper.
3.Add 100ul of Detection Reagent B working solution to each well. Incubate for 30 minutes at 37℃ after covering it with the Plate sealer.
4.Repeat the aspiration/wash process for total 5 times as conducted in step 2.
5.Add 90 ul of Substrate Solution to each well. Cover with a new Plate sealer. Incubate for 15-25 minutes at 37℃. Protect from light. The liquid will turn blue by the addtion of Substrate Solution.
6.Add 50 ul of Stop Solution to each well. The liquid will turn yellow by the addition of Stop solution. Mix the liquid by tapping the side of the plate. If color change does not appear uniform, gently tap the plate to ensure thorough mixing.
7.Remove any drop of water and fingerprint on the bottom of the plate and confirm there is no bubble on the surface of the liquid. Then, run the microplate reader and conduct measurement at 450nm immediately.

## Figures and Tables

**Table 1:** Baseline participant characteristics.

Variable	Control group (*n* = 30)	Intervention group (*n* = 32)
Gender		
Female, *n* (%)	11 (36.7)	13 (40.6)
Age, mean (SD)	54.00 ± 8.690	56.63 ± 7.088
Duration of hemodialysis, months, Md (Q1, Q3)	48.00 (20.50, 72.50)	27.50 (13.00, 75.00)
BMI	23.08 ± 1.286	22.96 ± 1.300
Kt/V	1.842 ± 0.02394	1.835 ± 0.02258
URR	71.44 ± 2.356	72.83 ± 3.304
Primary disease diagnosis		
Chronic glomerulonephritis	15 50	17 53.1
Diabetic nephropathy	7 23.3	8 25
Hypertensive nephropathy	5 16.7	5 15.6
Other primary diseases	3 10	2 6.3
Visual analogue scale	5.600 ± 2.127	5.750 ± 2.032
Calcium (mmol/L)	2.386 ± 0.3083	2.479 ± 0.3523
Phosphorus (mmol/L)	2.246 ± 0.6625	2.134 ± 0.5149
PTH (pg/L) Md (Q1, Q3)	239.0 (98.90, 435.5)	189.0 (92.90, 339.0)
Histamine (ng/mL) Md (Q1, Q3)	1.363 (0.524, 2.457)	0.897 (0.250, 2.349)
Substance P (pg/mL) Md (Q1, Q3)	10.81 (4.336, 22.59)	10.79 (5.094, 16.23)
PAR-2 (ng/mL) Md (Q1, Q3)	1.748 (0.828, 3.638)	1.927 (0.860, 3.884)
Tryptase (pg/mL) Md (Q1, Q3)	306.2 (148.7, 442.5)	371.1 (204.8, 524.0)

Md: median, Q1: 25% percentile, and Q3: 75% percentile.

*P* > 0.05.
